# Human hyaluronic acid synthase-1 promotes malignant transformation via epithelial-to-mesenchymal transition, micronucleation and centrosome abnormalities

**DOI:** 10.1186/s12964-017-0204-z

**Published:** 2017-11-14

**Authors:** Nguyet Nguyen, Awanit Kumar, Simi Chacko, Rodney J. Ouellette, Anirban Ghosh

**Affiliations:** 1 Atlantic Cancer Research Institute, 35 Providence Street, Moncton, NB E1C 8X3 Canada; 2 Department of Chemistry and Biochemistry, Université de Moncton, Moncton, NB Canada

**Keywords:** Hyaluronic acid Synthase-1, Malignant transformation, Epithelial-to-Mesenchymal transition, Genetic heterogeneity, Genomic instability, Micronucleus, Chromosomal instability and Centrosome abnormalities

## Abstract

**Background:**

Human hyaluronic acid (HA) molecules are synthesized by three membrane spanning Hyaluronic Acid Synthases (HAS1, HAS2 and HAS3). Of the three, HAS1 is found to be localized more into the cytoplasmic space where it synthesizes intracellular HA. HA is a ubiquitous glycosaminoglycan, mainly present in the extracellular matrix (ECM) and on the cell surface, but are also detected intracellularly. Accumulation of HA in cancer cells, the cancer-surrounding stroma, and ECM is generally considered an independent prognostic factors for patients. Higher HA production also correlates with higher tumor grade and more genetic heterogeneity in multiple cancer types which is known to contribute to drug resistance and results in treatment failure. Tumor heterogeneity and intra-tumor clonal diversity are major challenges for diagnosis and treatment. Identification of the driver pathway(s) that initiate genomic instability, tumor heterogeneity and subsequent phenotypic/clinical manifestations, are fundamental for the diagnosis and treatment of cancer. Thus far, no evidence was shown to correlate intracellular HA status (produced by HAS1) and the generation of genetic diversity in tumors.

**Methods:**

We tested different cell lines engineered to induce HAS1 expression. We measured the epithelial traits, centrosomal abnormalities, micronucleation and polynucleation of those HAS1-expressing cells. We performed real-time PCR, 3D cell culture assay, confocal microscopy, immunoblots and HA-capture methods.

**Results:**

Our results demonstrate that overexpression of HAS1 induces loss of epithelial traits, increases centrosomal abnormalities, micronucleation and polynucleation, which together indicate manifestation of malignant transformation, intratumoral genetic heterogeneity, and possibly create suitable niche for cancer stem cells generation.

**Conclusions:**

The intracellular HA produced by HAS1 can aggravate genomic instability and intratumor heterogeneity, pointing to a fundamental role of intracellular HA in cancer initiation and progression.

**Electronic supplementary material:**

The online version of this article (10.1186/s12964-017-0204-z) contains supplementary material, which is available to authorized users.

## Background

Hyaluronan or hyaluronic acid (HA) is a ubiquitous glycosaminoglycan mainly present in the extracellular matrix (ECM) and on the cell surface, but also present intracellularly where it is often associated with nucleoli and nuclear clefts [1–3]. HA is composed of repeating disaccharide units (N-acetylglucosamine and D-glucuronic acid), and in human HA molecules are synthesized by three Hyaluronic Acid Synthases [HAS1 (hCh19), HAS2 (hCh8), and HAS3 (hCh16)], each of which contains multiple transmembrane domains. Aberrant endogenous production of HA or treatment with exogenous HA in vitro has also been shown to promote cancer cell growth and malignant behavior in multiple model systems [4]. Overproduction of HA in breast carcinoma cells and in the surrounding stroma are considered independent prognostic factors for patient survival and correlate with higher tumor grade [1]. In addition, elevated serum HA levels are a hallmark of metastatic breast cancer [5–8].

Most of the studies that correlate overexpressed HA in cancer have been focused on HAS2 and HAS3 [9–12]. Among three isoenzymes, HAS2 and HAS3 have been shown to produce extracellular HA, whereas HA synthesized by HAS1 has been identified in both intra- and extracellular compartments of cancer cells [3, 9, 13]. HAS1 is mostly localized in the cytoplasmic space where it synthesizes intracellular HA [3] and appears to require a larger amount of substrate (UDP-N-acetyl glucosamine) [3] for the production of HA. The production of UDP-N-acetyl glucosamine is increased in all cancers due to observed dependence to the glycolytic pathway [14].

We and others have identified HAS1 as a key contributor to oncogenesis and disease progression in both hematological and solid cancers [13, 15–17]. Most of the previous studies on HA synthesized by HAS2 and HAS3 were focused mainly on its roles in the ECM and signal transduction, whereas the majority of HAS1 research has focused on prognostic marker studies or expression profiles of HAS1 genes in different cancers [3, 18–23]. In a recent retrospective histological study, authors found that a less favorable outcome of breast carcinoma patients is strongly associated with HAS1 expression (but not HAS2 and HAS3) as seen with shorter overall survival, higher relapse rate, estrogen receptor negativity and HER2 positivity [2]. In this study HAS1 was found to localize mostly in cytoplasm. HAS1 is also implicated in the growth and development of breast cancers, as well as the generation of intratumor heterogeneity [24] that maintains a cancer stem cell-like trait or phenotype [25]. HAS1 has been shown to be prognostic factor in multiple myeloma [18], colon cancer [17] and bladder cancer [19] and is overexpressed in a variety of other cancers [20–23].

Breast cancer and most other solid tumors display substantial cellular and genetic heterogeneity [26–30], which are used to establish clinical grade. Centrosome abnormalities and micronucleation are the prominent histological phenotypes of human cancers, including breast carcinoma [31–35]. Decades of histopathological observations lead to the hypothesis that centrosome abnormalities result in chromosomal instability (CIN) and that they have progressive involvement in advanced stages of carcinogenesis [31].

So far polysaccharide synthesis has not been mechanistically or molecularly correlated to causalities of carcinogenesis. Although ever-growing evidence indicates that the accumulation of intracellular and stromal HA during mammary carcinogenesis plays a role in cancer progression, a role for intracellular HA in genetic instability, generation of clonal diversity and cellular transformation in breast carcinoma or any other cancer, has not been reported [36]. Thus, the identification of a specific driver mechanism that generates centrosomal abnormality, genetic instability, micro-nucleus formation is critical for better understanding and treatment. Here we demonstrate a correlation between intracellular HA synthesized by HAS1 and the generation of clonal diversity and prevalence of genetic instability in cells. The results of this study suggests that overexpression of HAS1 induces loss of epithelial traits, centrosomal abnormalities, micronucleation and polynucleation, all of which are manifestations of malignant transformation. These observations reveal a previously unknown role of HAS1 in intracellular HA-mediated cellular transformation.

## Methods

### Cells, reagents and plasmids

MCF10A, HeLa and DLD1 cells were originally procured from the American Type Culture Collection (Manassas, VA) and grown in recommended culture media and conditions. MCF 10A, a non-tumorigenic mammary epithelial cell line is one of the widely used model to study loss of epithelial traits on 3D tissue culture. We chose DLD1 (colorectal adenocarcinoma) cell line, as 86% of population of these cells are diploid (46 chromosomes). Also the different cell lines were used to show the effect of HA overexpression in cell lines of diverse origin.

All the monoclonal antibodies were purchased from SantaCruz Biotech or Calbiochem. Anti-CD44 (F4 and DF1485), anti-BRCA1 (BR64 and D9), and GM130 (H7) antibodies were purchased from Santa Cruze Biotech. Anti-GFP (MAB2510) was from Calbiochem, and pericentrin antibody (ab4448–100) was from Abcam. Anti-A2 (a monoclonal antibody against A2-fusion-tag) was a kind gift from Prof. Greg Matlashewski, McGill University, Quebec, Canada). Transfection reagent Lipofectamine 2000 and RNA isolation reagent (TRIzol reagent) were from LifeTechnology. Biotinylated-hyalurone-binding-protein (bHABP) was purchased from Sigma. The mammalian HAS1 expression plasmids (pCDNA3 and pCEP4) were used from the previous published work [13]. Human HAS2 cDNA was purchased from OriGene. HAS1, HAS2 and GFP cDNAs were subcloned in pTRE2hyg or pTRE2pur vector from Clontech Laboratories, Inc. for conditional expression (tetracycline inducible). All the Tetracycline-inducible cell lines and their controls were subcultured in tetracycline-free media for maintenance. The tetracycline-on induction was done with doxycycline (Dox) in tetracycline-free media. The cell synchronization was done using double-thymidine-block method as described elsewhere [37]. Briefly, HeLa cells at 30% confluency was treated with 2 mM thymidine (final concentration) in culture media for 16 h, washed and incubated with normal media for 9 h. This was followed by a second thymidine treatment for 17 h. After second thymidine treatment the cells were grown in normal media for 10 more hours to collect for mitosis stage and 16 h to collect for G1/S stages.

### RT-qPCR

Total RNA was harvested with the TRIzol followed by RNeasy Kit (Qiagen) assisted isolation. One microgram of total RNA was used with the QuantiTect Reverse Transcription Kit (Qiagen) to synthesize cDNA. qPCR was performed using B-R SYBR Green SuperMix from iQuanta Biosciences using an Eppendorf Realplex2 Mastercycler. The ΔΔCt was calculated to identify fold change in gene expression normalized to GAPDH. Primers for E-Cadherin (5′-ATGCTGAGGATGGAGGTGGGT and 5′-CAAATGTGTTCAGCTCAGCCAGCA), N-Cadherin (5′-TGTGGGAATCCGACGAATGGATGA and 5′-TGGAGCCACTGCCTTCATAGTCAA), and GAPDH (5′-ACAGTCAGCCGCATCTTCTT and 5′ ACGACCAAATCCGTTGACTC) were used for qPCR.

### Transfection, selection and expression

The plasmid constructs were transfected to cells using Lipofectamine 2000 in 6-well tissue culture plates following manufacturer’s instructions. The cells were selected using appropriate antibiotic in tetracycline free media for two or 3 weeks followed by cultured into low antibiotic concentrations to maintain in tetracycline free media or frozen as cell-line-stock. We followed Clontech Laboratories manuals for TET-On expression induction system (pTRE2). In brief, for conditional expression of cDNA under tetracycline-responsive-elements, cells were first transfected and selected with pTET-On vector. The resulted populations were further transfected and selected with HAS1, HAS2 or GFP subcloned in pTRE2hyg and/or pTRE2pur for induced expression.

### Immunofluorescence microscopy and HA capture of active HAS1

For most of the immunofluorescence (IF) experiments cells were seeded onto 8-well chamber slides. The details of the IF methods were followed from our previous publication [13]. Briefly, the cells were fixed, permeabilized and blocked followed by overnight incubation with primary antibody or bHABP at 4 °C for protein or HA staining respectively. This was followed by incubation with secondary antibody and/or phalloidin conjugated to Alexa Fluor 594 Dyes (Molecular Probes, Invitrogen, Eugene, Oregon, USA) or streptavidin conjugated to Alexa Fluor 350 Dyes (Molecular Probes, Invitrogen, Eugene, Oregon, USA) in blocking solution for 1–2 h at room temperature, and finally mounted with PermaFluor aqueous mountant (Thermo Fisher Scientific, UK). We used Olympus FV1000 confocal microscope and used their image capture and analytical software (ASW2.1) for z-stacking, nuclear perimeter and fluorescent measurements for quantitation. For the capture of HA from actively synthesizing HAS1 and other HA-binding proteins, cell lysate were subjected to bHABP mediated HA-capture following the method described in our previous publication [13]. Briefly, cleared cell lysates were incubated with bHABP followed by incubation with streptavidin-sepharose beads (GE Healthcare) to collect HA bound to bHABP.

### 3D reconstituted cell-culture

A 3-dimensional cultures system was adopted where cells were seeded on top of the Matrigel. Matrigel™ Basement Membrane Matrix from BD Biosciences was used. Bottom of the cell culture plates (48-well) was layered with 50% Matrigel in MCF10A growth media and let them solidified as basement matrix. On that matrix 10,000 cells per well in 2% Matrigel with media (0.5 ml) were incubated. Twice every week half of the media was replaced with fresh media with 2% Matrigel. Cultures were monitored over 2 weeks for development of 3-dimensional ‘acini’ structures and photographed.

### Cell growth assay

Cell growth was monitored using the CellTiter-Blue® Cell Viability Assay Kit (Promega). Briefly, 5000 cells per well of 96 well plates were seeded in triplicate, and separate plates were used for each day of measurement. Different concentration of Doxycycline was added for induction of Tet-responsive genes as indicated. For measurements CellTiter-Blue® substrate was added, incubated for 1 h at 37 °C in 5% CO2 and fluorescence recorded at 560Ex/590Em. The fluorescence readings were normalized to day 0 of vehicle-control group and plotted as fold increased.

## Results

### Overexpression of human HAS1 increases intracellular HA

**Fig. 1:**
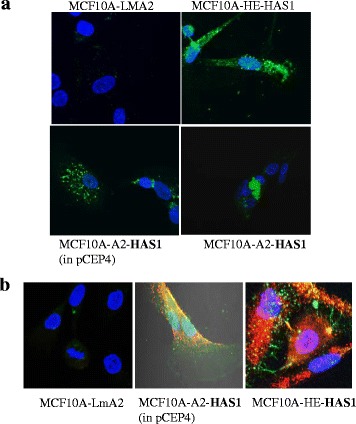
Overexpression of epitope-tagged human HAS1 increases cytoplasmic HA concentration in MCF10A cells. **a** Non-tumorogenic mammary cell line (MCF10A) expressing HAS1 shows significantly more HA staining (green) in comparison to protozoa-gene transfected MCF10A-LMA2 (control). The punctate localization of HAS1-synthesized HA inside the cell was observed. MCF10A-HE-HAS1: HAS1 in pCDNA3 with N-terminal hemagglutinin fusion-tag, MCF10A-A2-HAS1: HAS1 in pCDNA3 with N-terminal A2 fusion-tag, and MCF10A-LMA2: a *Leishmania major* (protozoa) gene (Lm2415) in pCDNA3 with C-terminal A2 fusion tag. The HAS1 in pCEP4 with N-terminal A2 fusion-tag is shown as ‘MCF10A-A2-HAS1 (in pCEP4). **b** MCF10A-A2-HAS1 and MCF10A-HE-HAS1 cells are brightly stained for HA (green) and CD44 (red) in comparison to MCF10A-LMA2 cells. The cells indicated in (A) were subjected to both HA and CD44 immunofluorescence staining. The middle panel shows the bright-field image to identify the edge of the cells. Nuclei were stained with DAPI (blue). Multiple focused- or Z-stacking images were used to compile these composite images for complete top-vision across the thickness of the cells

The normal mammary cell line MCF10A was transfected with plasmids that express HAS1 or an unrelated protozoan (*Leishmania major*) gene (LMA2) and were selected using appropriate antibiotic for 2 weeks. *Leishmania major* gene Lm2415 with A2 fusion tag (LMA2) has no homology with any mammalian gene, and hence we used as a control gene. It had the same A2 fusion-tag which was used to identify HAS1 expression as recombinant protein [13]. The selected populations of MCF10A cells were then seeded onto 8-well chamber slides, grown for 40 h and subjected to HA staining using biotinylated bovine HA binding protein (bHABP) and streptavidin-Alexa-488 fluorescent probes. MCF10A cells that express HAS1 with different mammalian expression plasmid backbones and fusion tags showed similar cytoplasmic localization of HA and significantly more HA staining in comparison to protozoa-gene transfected MCF10A (Fig. 1a). The punctate localization of the HA synthesized by overexpressed HAS1 inside the cell were observed in agreement with previously published results [1, 2, 13]. We also transiently transfected HAS1 into primary lung cells and observed cytoplasmic localization of synthesized HA (Additional file 1: Figure S1A). A similar pattern of intracellular HA expression was also observed in HeLa cells (Additional file 1: Figure S1B) and in DLD1 cells (Additional file 1: Figure S1C) with a tetracycline-inducible system of HAS1 expression, where the cells were transfected and selected for inducible cDNA expression: HAS1 in pTRE2-vector (puromycin). These data indicate that overexpression of HAS1 causes an increase in the expression of intracellular HA. We also evaluated whether the overexpression of HAS1 has any effect of cellular growth. We observed a lower mitotic index (Additional file 1: Figure S2A) for MCF10A cells that express HAS1 in comparison to LMA2-expressing or mock transfected MCF10A cells. Mitotic index is percentage of cells undergoing mitosis per 100 non-mitotic cells, and is a measure of the cellular growth rate. Similarly, as shown in the Additional file 2: Figure S2B, HeLa cells with tetracycline-inducible HAS1, non-induced background expression with 0 μg/ml Dox as well as 1, 3 and 6 μg/ml Dox inductions slowed the cell-population growth compared to the similar induction-scale of tetracycline-inducible GFP expressing cells. The higher the induction of the HAS1, the slower was the growth, with cells ceasing to grow at 6 μg/ml Dox induction on day 13 of culturing.

CD44 and its splice-variants are well-characterized HA receptors that are implicated in cellular transformation, as well as being a stem cell marker [24, 38–41] we therefore sought to determine whether overexpression of HAS1 and the resulting increase in cytoplasmic HA is related to concomitant increase in cytoplasmic CD44 expression and/or localization. To answer this we transfected MCF10A cells, selected and seeded onto 8-well chamber slides for HA- and immuno- staining. As shown in the Fig. 1b the expression of CD44 in the cytoplasm was significantly higher in the HAS1 overexpressing cells than in the control LMA2-expressing MCF10A cells, and HA and CD44 were found to be co-localized in many areas of the cell. These results indicate that cytoplasmic overexpression of HA, synthesized by HAS1, concomitantly increased the endogenous expression of CD44 in the cell.

### Overexpression of HAS1 induces epithelial-to-Mesenchymal transition (EMT) in MCF10A cells

**Fig. 2:**
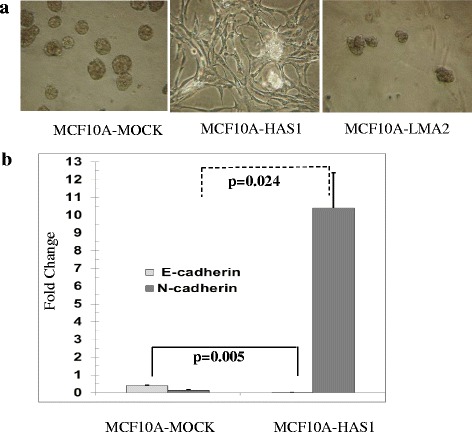
Overexpression of HAS1 induces Epithelial-to-Mesenchymal Transition (EMT) in MCF10A cells. **a** The normal mammary cell line MCF10A was mock transfected (left), or transfected with a plasmids that express HAS1 (middle) or an unrelated protozoa gene LMA2 (right). The selected populations were cultured in 3D reconstituted basement-membrane model. Both control panels (right and left) show acini structure in contrast to middle panel. **b** MCF10A cells were transfected as described in (a) and subjected to EMT analysis using the common EMT markers E-cadherin (Epithelial) and N-cadherin (Mesenchymal) using RT-qPCR. The relative expression of E-cadherin is diminished, however N-cadherin is over-expressed in MCF10A-HAS1 cells

EMT is one of the hallmarks of cellular invasiveness / transformation and widely regarded as phenotype of cancer progression. As HAS1 is a prominent prognostic factor in breast cancer and other cancers [2, 17–25] we sought to determine whether overexpression of HAS1 influences EMT and thereby skew the cellular fate. MCF10A cells grown in 3D culture produce mammary epithelial acini-mimicking structures; however, in contrast the induction of EMT in MCF10A cells causes a diffuse network of cells without any 3D structure [42]. MCF10A cells were mock transfected (no plasmid) or transfected with a plasmid that expresses HAS1 or an unrelated protozoa gene (LMA2). The selected cell populations were subjected to reconstituted basement membrane 3D culture using Matrigel. Mock-transfected or protozoa-gene-transfected MCF10A cultures developed acini structures characteristic of the epithelial nature of the cells (Fig. 2a) whereas MCF10A cell-population selected with HAS1 distinctively did not produce any acini structures, but rather showed a loose cellular network, characteristic of transformed mesenchymal-type cells. Figure 2a is representative of four independent experiments. To identify EMT gene-expression signatures we performed quantitative RT-PCR analysis of E-cadherin (epithelial) and N-cadherin (mesenchymal) in HAS1-expressing cells as well as control populations. Quantification of E-cadherin and N-cadherin transcripts was performed using RT-qPCR and GAPDH (Glyceraldehyde 3-phosphate dehydrogenase) was used as normalization control for ΔΔCt measurements. The quantitation of the fold increase was measured by 2^-ΔΔCt^ (where ΔΔCt = ΔCt of sample - ΔCt of GAPDH). Relative expression of E-cadherin transcript was significantly diminished in HAS1-expressing cells (*p* = 0.005) whereas the expression of N-cadherin increased by almost 10-fold in HAS1-expresing cells (*p* = 0.024), suggesting that MCF10A cells that overexpressed HAS1 induced EMT (Fig. 2b). The data is representative of three experiments (triplicate per sample per experiment).

### Prolonged HAS1 expression induces clonal diversity in cell-population

**Fig. 3:**
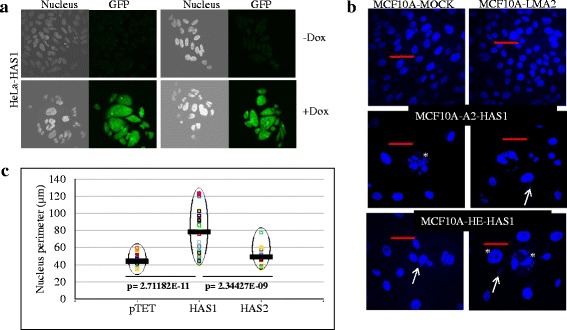
Prolonged expression of HAS1 increases clonal diversity. **a** A spectrum of clonal diversities was observed in the Dox-induced populations (lower panels) varying widely in nuclear/cytoplasmic sizes and shapes. The Dox induction was verified with higher GFP expression in +Dox cells (lower panel) then the –Dox controls (upper panel). This figure is representative of at least 5 individual induction experiments. **b** MCF10A cells were transfected with plasmids as indicated in Fig. 1a. HAS1 transfected cells show variation of the nuclear size and shapes demonstrating clonal variations. The resulted cell population were found homogenous in control mock transfected (upper left) as well as irrelevant control protein expressing (LMA2) cells (upper right). White arrows show micro-nucleus, asterisk indicate multi-nucleated or huge donut-shaped nucleus, and the red bar is 50 μm. **c** DLD1 colorectal adenocarcinoma cell line was transfected and selected with Tet-inducible HAS1 or HAS2 or control (pTET) systems, fixed and stained the nucleus with DAPI (blue). HAS1 and HAS2 cells showed much diversity in nuclear perimeter size in comparison to pTET cells (control). The cells were observed under confocal microscope and photographed for multiple fields from each slides. The nuclear perimeter (without micronucleus) were marked and measured with Olympus FV1000 software. The average perimeter is marked with thick horizontal bars. The range of nuclear size/morphological diversity is shown in dashed elliptical areas

Intra-tumor clonal diversity as a measure of cancer progression and drug resistance is well studied [28, 43]. However, correlation of clonal diversity (and aneuploidy) induced by polysaccharide synthesis has not been described. We observed these strikingly unique effects in the progeny cell populations that ectopically express HAS1 for several generations. Transient overexpression of HAS1 for 40 to 72 h did not produce a cell population with differing cellular and nuclear morphologies (data not shown) [13]. As shown in Fig. 3a, a diverse and heterogeneous population emerged when HeLa cells were induced (+Dox) to express HAS1 for a prolonged period (10 weeks, 3 μg/ml Dox), however the cells were morphologically similar to each other in the non-induced (−Dox) population. These cells were also engineered to co-express GFP under tetracycline responsive promoters to verify conditional expression of GFP due to induction with Dox and to identify any background (leaky) expression of this tetracycline-inducible plasmid system without any Dox induction. Similarly we also observed high population diversity in nuclear morphology (Fig. 3b) when HAS1 was expressed under a CMV promoter continuously for 10 weeks after transfection and selection in MCF10A cells compared to mock-transfected cells that express an unrelated protozoa gene (LMA2).

We also tested DLD1 cells (colorectal adenocarcinoma cell line, 46 chromosomes occurring in 86% of population) for the effects of HAS1 overexpression using a tetracycline-inducible system and observed similar outcome of emergent population’s diversity (shown as nuclear perimeter) when HAS1 was overexpressed (4 weeks induction with 3 μg/ml of Dox), but no effects on nuclear morphology in control cells (pTET) or cells that overexpressed the related HAS2 (Fig. 3c). These results indicate that expression of cytoplasmic HA synthesized by HAS1 induces population diversity irrespective of cell types and expression systems.

### HAS1 expression induces micronucleus formation

**Fig. 4:**
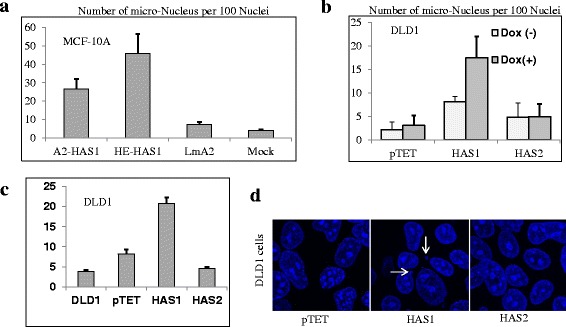
HAS1 expression induces micronucleation. **a** A2-HAS1 and HE-HAS1 has more micro-nuclei than LMA2 and Mock transfected MCF10A cells. The average number of micronucleus / 100 nuclei from the cells are presented from 6 microscopic fields (40×) with respective standard errors. **b** HAS1 [but not pTET (control) or HAS2 cells] induced micronucleation in transfected DLD1 cells with short-term induction. Average numbers of micronucleus/100 nuclei are presented with standard error bar. **c** The long-term (4 weeks) culture of Dox-induced cells (from Fig. B) continue to produce micronuclei only when HAS1s were expressed but not for cell alone (DLD1, maintained in Tet-free media for 10 weeks), pTET-control cells or HAS2 transfected cells. Average number of micronucleus / 100 nuclei are presented with standard error bar. **d** Sample photographs of Tet-inducible DLD1-cells induced for 100 h. The micronuclei were observed in HAS1 expressing cells only (middle panel) and indicated with arrow-heads

Cytogenetically the progression of cancer is driven by the generation of population diversity, micronucleus formation and chromosomal instability which generate aggressive clones, drug-resistance phenotypes, and the emergence of cancer stem cells [44, 45]. We observed that long-term HAS1-expressing cells produce morphologically divergent cells with an abnormally high incidence of micronucleated and polynucleated cells, possibly indicating ongoing generation of aneuploidy and continued chromosomal instability. As demonstrated in Fig. 4a, the mock- and LMA2-transfected and selected (6 to 7 weeks after transfection) population of MCF10A cells have 5–10 μ-nuclei per 100 nuclei, whereas in HAS1-transfected cells have 30 to 50 micronuclei per 100 nuclei. We further verified the above HAS1-associated phenomenon in tetracycline-inducible DLD1 cells, which conditionally expressed HAS1 and HAS2. Expression of HAS1 and HAS2 was induced with Dox for short-term (100 h, Fig. 4b) as well as long-term (4 weeks, Fig. 4c) to examine micronucleus formation. As depicted in Fig. 4b DLD1 cells reproduced the phenomenon of micronuclei formation upon short-term HAS1 expression (*p* = 0.078), but micronuelci were not observed in HAS2-expressing (*p* = 0.96) and control DLD1 (pTET) (*p* = 0.71) cell populations. Cells that contain the inducible HAS1 plasmid showed a significant increase in micronuclei formation over control and HAS2 cells in the absence of Dox induction due to leaky HAS1 expression, corroborating the fact that minimal HAS1 expression (but not HAS2) induces micronuclei formation (Fig. 4b). Representative photographs of short-term Dox induction is shown in Fig. 4d. A similar pattern of micronuclei formation was observed for long-term HAS1 induction (4 weeks) in DLD1 cells (Fig. 4c). The rate of micronucleation did not significantly differ in short- verses long-term induction of HAS1, possibly indicating sustained chromosomal instability once cytoplasmic HA levels were increased by HAS1 expression.

### HAS1 expression compromises centrosome integrity

**Fig. 5:**
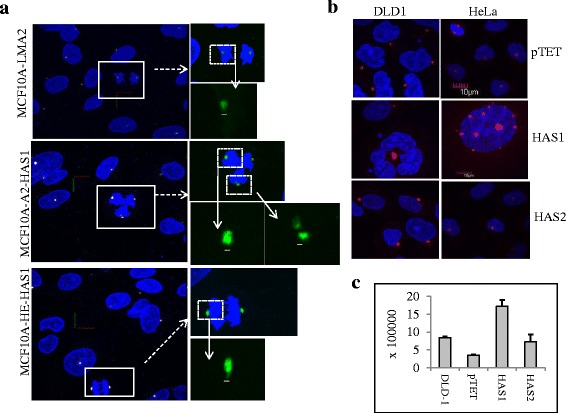
HAS1 expression induces centrosomal abnormalities. **a** MCF10A cells transfected with indicated plasmids were selected followed by centrosome staining using an antibody against pericentrin. The nucleus was stained with DAPI (blue). The left column shows the composite image of pericentrin (white) and nucleus (blue). The red and green scale bars on the left-panels are 10 μm each. The right-top-panels show zoom-in of the indicated area from the left panels (pericentrin as green). Bottom right panels are the magnification of the indicated dotted area from the right-top panels to visualize only pericentrin staining (green). The white bar is 1 μm in lower right panels. **b** Representative images of centrosomal abnormalities in DLD1 cells (left panel) and HeLa cells (right panel). Cells expressing HAS1 (middle panels) for both cell lines show centrosomal abnormalities but no such event was observed in pTET (upper panels) and HAS2 (lower panels) expressing cells. The cells population were immunostained for pericentrin (red) and the nucleus stained with DAPI (blue). Tetracycline-inducible lines (DLD1 and HeLa) were passaged though continuous Dox exposure to induce HAS1 and HAS2 mediated HA synthesis for many generations (10 weeks). The pTET cells were used as controls. **c** The average fluorescence intensity of pericentrin staining per nucleus was calculated from at least four random regions of interest (ROI) and represented with the standard errors. The DLD1 cells expressing HAS1 has higher intensity than DLD1 cells alone, pTET and HAS2 expressing cells. Relative fluorescent intensity of the red channel (pericentrin) from the confocal images was collected for ROI and the total number of nucleus was counted. Multiple focused- or Z-stacking images were used to compile these composite images for complete coverage across the thickness of the cells

Mitotic aberrations are the result of many possible mitotic abnormalities. Most cells with mitotic aberrations die off from the population, but cancer cells inherently sustain signatures of mitotic aberrations to generate clonal diversity. One of the most predominant mitotic aberrations is caused by centrosome abnormalities (amplification by number and volume), which are correlated to cancer progression [46]. Centrosome abnormalities and a high incidence of micronuclei are considered hallmark signatures of chromosomal instability and are found in all solid tumors, including breast cancer [47–50]. Here we tested whether cells with HAS1-synthesized cytoplasmic HA, which resulted in clonal diversity and micronucleation, could also display signatures of centrosomal abnormalities. Immunofluorescence staining of centrosomes with anti-pericentrin antibody revealed enlarged, fragmented or multipolar centrosomes in HAS1-expressing MCF10A populations but not in control LMA2-expressing cells (Fig. 5a). Pericentrin is integral to the filamentous matrix of the centrosome and is involved in the initial establishment of organized microtubule arrays of the mitotic apparatus. A similar pattern of centrosome abnormalities were also observed in tetracycline-inducible HAS1-expressing HeLa and DLD1 cells, but not in control (pTET) and HAS2-expressing populations (Fig. 5b). To quantitate the enlargement of centrosomes (Fig. 5c) we extracted fluorescence intensity from the region of interest (RIO) of anti-pericentrin staining (red channel) from multiple fields of confocal images collected from DLD1 cells. The ROI fluorescent units of pericentrin represent the comparative volume and number of centrosomes, which was significantly increased in HAS1-expressing cells (Fig. 5c). We also observed a comparatively larger Golgi apparatus when cells undergo HAS1-induced centrosome abnormalities (Additional file 3: Figure S3A). The above observations of centrosome abnormalities caused by HAS1 expression in multiple cell types confirm the association of HAS1 expression with centrosome abnormalities.

### RHAMM and BRCA1 interact with HAS1-synthesized cytoplasmic HA

**Fig. 6:**
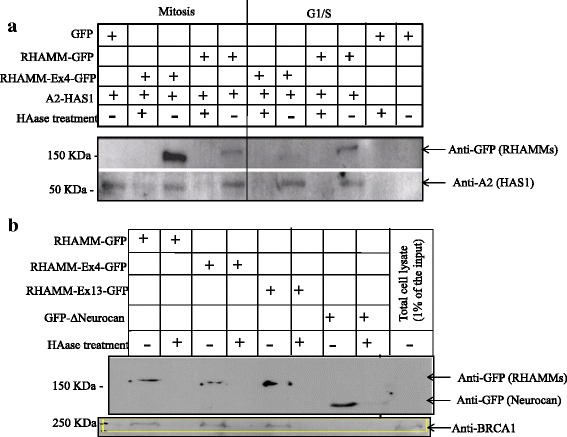
HAS1-synthesized HA interacts with RHAMM. **a** RHAMM and its splice variants are associated with cellular HA (synthesized from HAS1 overexpression) during mitosis and G1/S phase. HeLa cells were co-transfected with plasmids expressing A2-tagged HAS1 (A2-HAS1) and full-length GFP-tagged RHAMM (RHAMM-GFP) or the splice-variants of RHAMM (RHAMM-Ex4-GFP). Cell populations were synchronized in Mitosis or G1/S using thymidine block and synchronization was verified using flow cytometry (Supplementary Fig. 3B). Total cellular HA was isolated using biotinylated bovine HA-binding-protein and streptavidin-conjugated magnetic beads. The isolated beads were treated (+) with hyaluronidase (HAase). Samples were subjected to immunoblotting for RHAMM and HAS1. **b** BRCA1 interacted with RHAMM isoforms but not with the other HA-binding protein Neurocan. GFP-tagged RHAMM isoforms and GFP-ΔNeurocan were expressed in HeLa cells and HA-binding proteins were isolated from the cell lysates using biotinylated-HA as “bait”. Immunoblotting of pull-down material with and without HAase treatment revealed that endogenous BRCA1 were found to be associated with RHAMM isoforms (but not with Neurocan), suggesting that BRCA1 may interact directly with RHAMM

Hyaluronan-mediated motility receptor (RHAMM), also known as CD168, is an HA-binding protein and has been implicated in mitotic spindle formation/stability, correlated with poor outcomes in many cancers [51–54], and is implicated in metastasis [55, 56]. Our results correlating micronucleus generation and centrosome abnormalities with HAS1 expression prompted us to investigate the possible molecular interactions between spindle-centrosome assembly and cytoplasmic HA produced by HAS1. The dynamics of proper mitotic spindle machinery formation, stability and segregation relies on many proteins (for example RHAMM) [57]. RHAMM possesses partially overlapping centrosome-binding and HA-binding domains [42], which may provide a potential clue as to how cytoplasmic HA interferes with spindle formation. Naturally occurring RHAMM splice variants that lack exon 4 and exon 13 preserve the HA-binding domain [58]. To determine whether HA that is synthesized by HAS1 interacts with RHAMM, HeLa cells were transiently co-transfected with plasmids that express HAS1 along with GFP-tagged full-length RHAMM or a splice-variant of RHAMM (exon 4). The transfected cells were synchronized for Mitosis or G1/S cell cycle stages using thymidine block, which was verified by flow cytometry (Additional file 3: Figure S3B). The cells were synchronized in mitosis (when the nuclear membrane is dissolved) because at this stage cytoplasmic HA may have a higher chance of interaction with the mitotic machinery. Total cellular HA was captured using biotinylated bovine HA-binding-protein (bHABP, Sigma) and streptavidin -conjugated magnetic beads from the indicated cell lysates (Fig. 6a). Half of the captured material was treated with hyaluronidase (HAase, an HA degrading enzyme) to remove any HA or its bound proteins. Both the HAase-treated and untreated samples were subjected to immunoblotting for RHAMM and HAS1. De novo synthesized HA by HAS1 is covalently attached to HAS1 during its elongation [13], therefore HAS1 and any protein(s) bound to HA can be isolated using bHABP [13]. As shown in Fig. 6a, we observed a differential, but specific, association of RHAMM and its splice variant with cellular HA during mitosis and G1/S phase. Neither RHAMM nor HAS1 were detected when the captured material was treated with HAase, indicating both of these proteins are associated through cellular HA. This result suggests that de novo HA synthesized by HAS1 interacts with RHAMM during mitosis, as well as G1/S phases of cell cycles, possibly at different ratios.

Cytoplasmic RHAMM also interacts with BRCA1 [59] irrespective of the cell-cycle stage. BRCA1 is one of the most researched breast and ovarian cancer susceptibility gene that has been found to be associated with γ-tubulin of centrosome [60] and thus may act as an essential component of spindle formation. We wanted to explore whether HA-interacting RHAMM also associates with the BRCA1 protein in these cells. GFP-tagged RHAMM full-length and its splice variants (exon 4 and exon 13) were transiently expressed in HeLa cells and cell lysates were incubated with biotinylated-HA as bait, followed by streptavidin-bead-mediated capture. We also expressed GFP-ΔNeurocan in HeLa cells as control. Neurocan is an HA-binding protein and the HA-binding domain of Neurocan is fused with GFP in the GFP-ΔNeurocan expression construct [61]. The captured material (HA-binding proteins) was digested with HAase to confirm that the captured HA-associated proteins are directly or indirectly associated with biotinylated-HA. Immunoblot analysis showed that the GFP-RHAMM proteins and GFP-ΔNeurocan were bound by the ‘bait’ HA (Fig. 6b). The same immunoblot was reprobed with anti-BRCA1 antibody to identify the association of endogenous BRCA1 with HA-captured proteins. BRCA1 was found to be associated with the HA-RHAMM complex, but not with Neurocan-HA complex, suggesting that BRCA1 may interact directly with RHAMM as BRCA1 does not possess an HA-binding domain. These results demonstrate the molecular interactions of HAS1-synthesized cytoplasmic HA with RHAMM and possibly with BRCA1, which are essential for proper spindle as well centrosomal functions.

## Discussion

Hyaluronic acid (HA) is composed of repeating disaccharide units and is synthesized by three different membrane-bound synthase enzymes (HAS1, HAS2 and HAS3). HA is present ubiquitously in the extracellular matrix and undergoes rapid turnover. HAS1 expression is typically very low in healthy cells [3, 9, 13, 62, 63] and found to be upregulated in specific inflammatory conditions like osteoarthritis [64]. Histology and in vitro overexpression data suggest that HAS1 accumulates significantly more in the cytoplasmic space rather than on the plasma membrane [1, 2, 13, 65, 66]. Enzymatically-active splice variants of HAS1 exclusively accumulate in the cytoplasm and have the ability to retain the full-length HAS1 in the cytoplasm because HAS1 multimerizes for its proper enzymatic function [13].

Our results demonstrate that an increase in cytoplasmic HA through overexpression of HAS1 in different human cell types increases the characteristic cancer phenotypes of clonal variation, EMT, micronucleation and centrosome abnormalities such as clustering and/or fragmentation. Published reports characterizing HAS3 did not describe any such cytoplasmic HA production by HAS3 and its related effects [11, 67]. HAS2 and HAS3 were found to produce extracellular HA [66], and HAS2 appears to contribute to cytoplasmic HA production when HAS2 is overexpressed in pancreatic cancer cells [11]. Though HAS2 expression is correlated with EMT [68, 69], neither HAS1 nor HAS3 have been directly implicated in EMT. To our knowledge, no reports are found correlating neither HAS2 nor HAS3 with clonal, variation, micronucleation or centrosome abnormalities. Thus, our results demonstrate unique HAS1-specific phenotype (clonal variations, micronucleation and centrosome abnormalities), which are not evident upon HAS2 overexpression. The results presented here are reproducible in non-cancer cells (MCF10A) and as well as with different cancer cell lines (cervical and colon) as model systems. Our data support the hypothesis that HAS1-synthesized cytoplasmic HA correlates with the interference in genomic stability and normal mitosis.

Centrosome abnormalities and CIN manifest in cells as aneuploidy and/or micronucleation and often leads to decreased survival, however surviving sub-clones may suggest the emergence of therapy-resistant clones or tumor stem cells [47, 70, 71]. Centrosome abnormalities and/or micronucleation are the most commonly-detected markers in most human cancers (solid and hematological) [33, 47]. Cell cycle, mitosis, DNA repair, proliferation and tissue apicobasal polarity depend on precise centrosome divisions and localization [47]. The integral relation of centrosome abnormality, its spatial association with the Golgi and overall role in CIN has been explored by many groups, who have sought to find the mechanistic and molecular interactions driving cancer initiation and development (reviewed in [72]). Nevertheless, how CIN contributes to cancer is not well understood, notwithstanding the fact that the presence of micronuclei indicates the extent of CIN progression, eventually increasing the probability of clonal diversification leading to cellular transformation [45, 73]. Surviving cancer cells that possess clustered/enlarged centrosomes and micronuclei may undergo abnormal cell divisions, risking further genetic instability that contributing to EMT, cancer progression and cancer stem cell initiation [47]. Our findings demonstrate for the first time that cytoplasmic HA homeostasis is required to maintain precise centrosome functions and to prevent both EMT, and micronucleation and their subsequent effects on cellular fate.

We found expression of CD44 in cytoplasm and also to some extent on the plasma membrane in MCF10A cells only when HAS1 was expressed (Fig. 1b). This may indicate CD44-mediated induction of EMT (as in Fig. 2) and may correlate with CD44 associated cancer stem cell initiation [41]. Therefore we do not rule out the possibility of CD44-expression mediated EMT. The exact mechanism(s) that correlate CD44 with the observed abnormalities prompt merit for further investigation.

Both full-length and splice variants of HAS1 and RHAMM are overexpressed in multiple myeloma and bladder cancers, but absent in healthy cells [18, 39, 74]. RHAMM is a cytoplasmic protein that is unconventionally exported to the cell surface during wound repair, where it binds with HA and activates CD44 resulting in stimulation of the Ras/Erk1,2 pathway, a cascade that functions in cellular proliferation and that often dysregulated in cancer [40]. The centrosomal-targeting domain of RHAMM overlaps with its HA-binding domains [58]. Intracellular RHAMM is found in multiple compartments, but its association with the centrosome and mitotic spindle are the best characterized [42]. RHAMM/centrosome/spindle interactions are required for normal spindle formation and for passage through the G2/M stage of the cell cycle [57]. RHAMM, Aurora kinases and Polo-like kinase-1 are centrosome-associated proteins that have important roles in cell cycle progression, checkpoint control and mitosis. TPX2, a spindle assembly factor, mediates AuroraA kinase (AURKA) localization to spindle microtubules and activates it by autophosphorylation [57]. Recent studies also showed that BRCA1, RHAMM, AURKA and TPX2 interactions are mechanistically important for microtubular reorganization during mitotic spindle formation and apicobasal polarization for tissue organization [75]: all these interactions highlight the critical and precise functions of centrosome. These fundamental aspects of centrosome functions are compromised in most malignancies, and our results suggest that this could be due to an imbalance in cytoplasmic HA.

Previous work on HA has mainly focused on its ECM-related and signal transduction roles. So far, no studies have reported any correlation between cytoplasmic HA imbalance, micronucleation and centrosome abnormalities in any cell type. A previously unknown role for cytoplasmic HA that HAS1-synthesized cytoplasmic HA induce EMT, micronucleation, centrosome abnormalities, and aneuploidy in different cell types is supported with these results. Although growing evidence indicates accumulation of cytoplasmic HA during mitosis [76], a role for cytopalsmic HA in genetic stability and cellular transformation has not yet been reported.

## Conclusions

Using different cells and expression systems as models, we demonstrated that HAS1 expression induced clonal diversity, multi-nucleus formation, micronucleus generation, centrosome abnormalities which are the most common cancer-associated cellular anomalies found in histological studies. Our results also indicate that intracellular HA produced by HAS1 is associated with the BRCA1-RHAMM-microtubule complex, which implies a possible mechanistic role in cancer initiation and progression. Overall, our findings that HA, a primitive glycosaminoglycan influences EMT, centrosome abnormalities, and micronucleation noteworthy and will contribute to a better understanding of cancer biology and may eventually lead to clinical and therapeutic opportunities.

## Additional files

Additional file 1: Figure S1. Expression of HAS1. **(A)** Lung primary cells were transiently transfected with pCDNA3-A2-HAS1 or empty vector (pCDNA3-A2) and subjected to HA fluorescence staining (green) after 72 h. Nucleus was stained with DAPI. **(B)** HeLa cells were engineered and selected for Tetracycline-on inducible HAS1 expression. Cells were grown in tetracycline-free media in 8-well chamber slides for 16 h followed by with or without doxycycline (Dox) treatment for 40 h, and then HA fluorescence staining (white) and nuclear staining with DAPI (blue). **(C)** DLD1 cells were transfected and selected for Tet-inducible HAS1 expression. The cells were grown in tetracycline-free media followed by induced with doxycycline (Dox) treatment for 40 h. The cells were stained for HA localization using bHABP (Green). DLD1-pTET cells served as negative control. (PDF 150 kb)

Additional file 2: Figure S2. Effect of HAS1 expression on mitotic index and cell growth. **(A)** Lower mitotic index was observed in HAS1 expressing MCF10A cells in comparison to LMA2-expressing of mock transfected cells. MCF10A cells transfected with the indicated cDNA in pCDNA3. The selected populations were seeded onto 8-chamber glass slides, incubated overnight, and then fixed and DAPI-stained to count mitotic/non-mitotic nuclei based on the chromatin / nucleus structure. **HE-HAS1**: HAS1 in pCDNA3 with N-terminal hemagglutinin fusion-tag, **A2-HAS1**: HAS1 in pCDNA3 with N-terminal A2 fusion-tag, **LMA2**: unrelated protozoa gene in pCDNA3 with C-terminal A2 fusion tag and **Mock**: transfection without any plasmid and not selected with any antibiotic. **(B)** HAS1 expressing cells showed the slower growth after induction with Dox. HeLa cells engineered and selected for Tetracycline-on inducible HAS1 or GFP expressing plasmids. The cell populations were subjected to growth analysis to test the effect of inducible expression of genes (GFP and HAS1) on growth for 13-days with Dox at different concentrations. The results are presented as fold increase of viable cells compared to seeded cells at Day 0. The growth of all HAS1-expressing cells was slower than the GFP-puromycin-vector controls, may be due to background synthesis (leakiness) of intracellular-HA by HAS1 even at 0 μg/ml Dox induction. At higher concentrations of Dox (6 μg/ml) the growth cease beyond 10th day for HAS1 but not for control GFP. (PDF 12 kb)

Additional file 3: Figure S3. **(A)** Larger Golgi apparatus were observed in the cells expressing HAS1 (lower panels) as compared to control pTET cells (upper panels). The tetracycline-inducible DLD1 cells with HAS1 and control (pTET) as described in Fig. 5B were stained for Golgi bodies (GM130, green), centrosome (pericentrin, red) and nucleus (blue) in the first panel, and HA (white) in the second panel and DIC image of the structure of the cell in third panel. **(B)** Respective cell populations indicate the synchronized cells at mitosis and G1/S phase of the cell cycle. Transfected HeLa cells were synchronized with double thymidine blocks. The cells were measured for their DNA contents using flow cytometry to verify synchronization. The cells were harvested, fixed with cold ethanol and stained with propidium iodide to measure the content of DNA in cell-populations. (PDF 158 kb)

## Abbreviations

AURKA: AuroraA kinase
bHABP: Biotinylated-hyalurone-binding-protein
CIN: Chromosomal instability
Dox: Doxycycline
ECM: Extracellular matrix
EMT: Epithelial-to-Mesenchymal Transition
HA: Hyaluronic acid
HAase: Hyaluronidase
HAS1: Hyaluronic Acid Synthases 1
HAS2: Hyaluronic Acid Synthases 2
HAS3: Hyaluronic Acid Synthases 3
IF: Immunofluorescence
RHAMM: Hyaluronan-mediated motility receptor
ROI: Region of interest
