# Supplementary figures and images for: Human hyaluronic acid synthase-1 promotes malignant transformation via epithelial-to-mesenchymal transition, micronucleation and centrosome abnormalities
Source: Cell Commun Signal. 2017 Nov 14;15:48. doi: 10.1186/s12964-017-0204-z (PMC5686803; doi:10.1186/s12964-017-0204-z)

## Supplementary figure 1

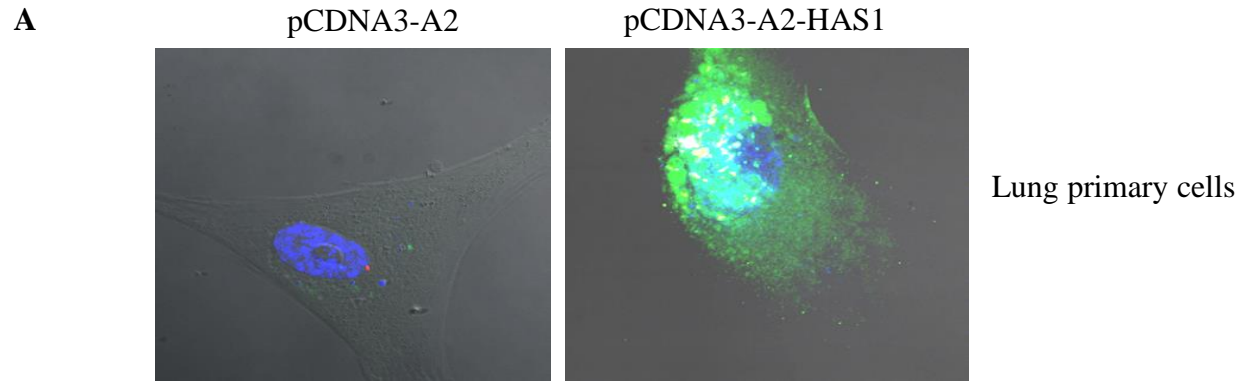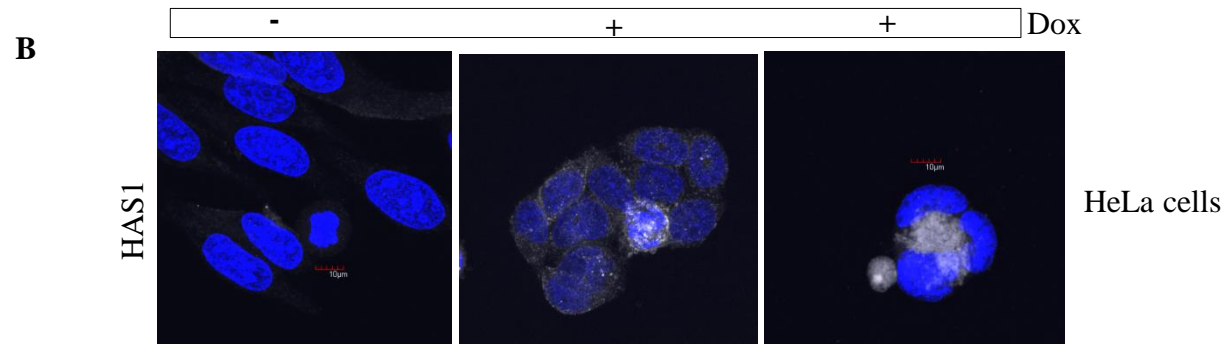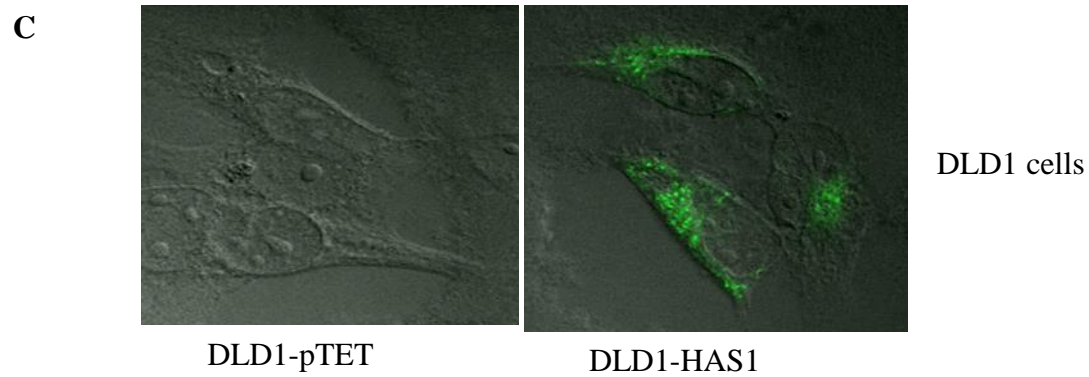

Supplement: Supplementary file 1 — Expression of HAS1. (A) Lung primary cells were transiently transfected with pCDNA3-A2-HAS1 or empty vector (pCDNA3-A2) and subjected to HA fluorescence staining (green) after 72 h. Nucleus was stained with DAPI. (B) HeLa cells were engineered and selected for Tetracycline-on inducible HAS1 expression. Cells were grown in tetracycline-free media in 8-well chamber slides for 16 h followed by with or without doxycycline (Dox) treatment for 40 h, and then HA fluorescence staining (white) and nuclear staining with DAPI (blue). (C) DLD1 cells were transfected and selected for Tet-inducible HAS1 expression. The cells were grown in tetracycline-free media followed by induced with doxycycline (Dox) treatment for 40 h. The cells were stained for HA localization using bHABP (Green). DLD1-pTET cells served as negative control. (PDF 150 kb) [file 12964_2017_204_MOESM1_ESM.pdf]

## Supplementary figure 2

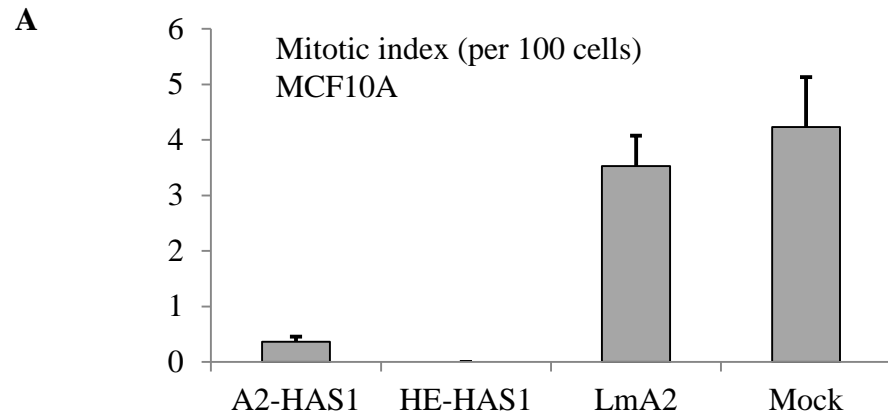

**B**

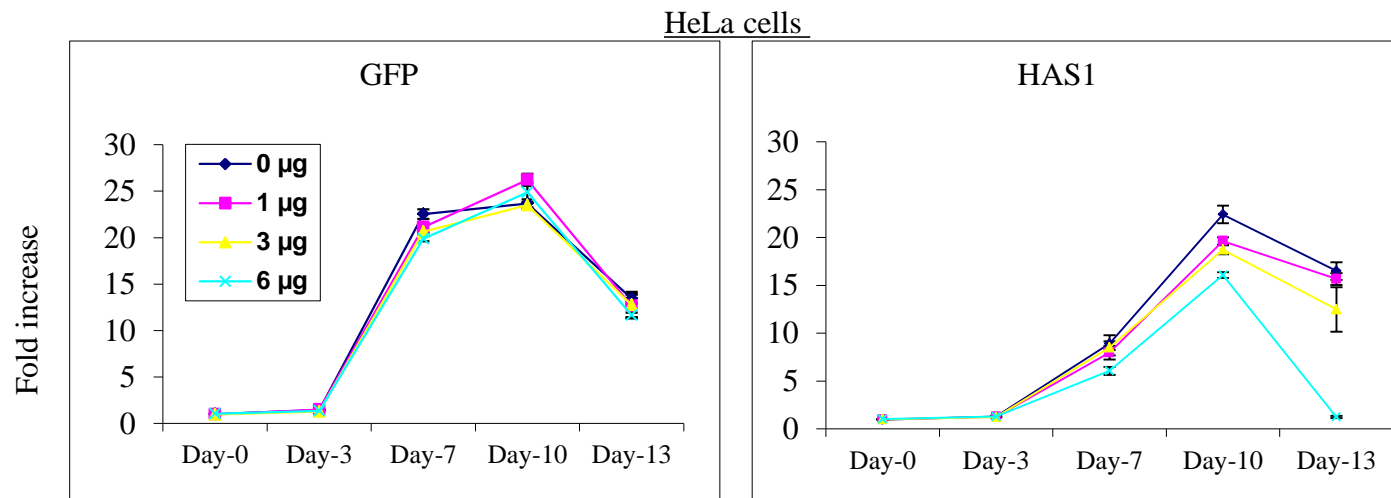

Supplement: Supplementary file 2 — Effect of HAS1 expression on mitotic index and cell growth. (A) Lower mitotic index was observed in HAS1 expressing MCF10A cells in comparison to LMA2-expressing of mock transfected cells. MCF10A cells transfected with the indicated cDNA in pCDNA3. The selected populations were seeded onto 8-chamber glass slides, incubated overnight, and then fixed and DAPI-stained to count mitotic/non-mitotic nuclei based on the chromatin / nucleus structure. HE-HAS1: HAS1 in pCDNA3 with N-terminal hemagglutinin fusion-tag, A2-HAS1: HAS1 in pCDNA3 with N-terminal A2 fusion-tag, LMA2: unrelated protozoa gene in pCDNA3 with C-terminal A2 fusion tag and Mock: transfection without any plasmid and not selected with any antibiotic. (B) HAS1 expressing cells showed the slower growth after induction with Dox. HeLa cells engineered and selected for Tetracycline-on inducible HAS1 or GFP expressing plasmids. The cell populations were subjected to growth analysis to test the effect of inducible expression of genes (GFP and HAS1) on growth for 13-days with Dox at different concentrations. The results are presented as fold increase of viable cells compared to seeded cells at Day 0. The growth of all HAS1-expressing cells was slower than the GFP-puromycin-vector controls, may be due to background synthesis (leakiness) of intracellular-HA by HAS1 even at 0 μg/ml Dox induction. At higher concentrations of Dox (6 μg/ml) the growth cease beyond 10th day for HAS1 but not for control GFP. (PDF 12 kb) [file 12964_2017_204_MOESM2_ESM.pdf]

Supplementary figure 3

A

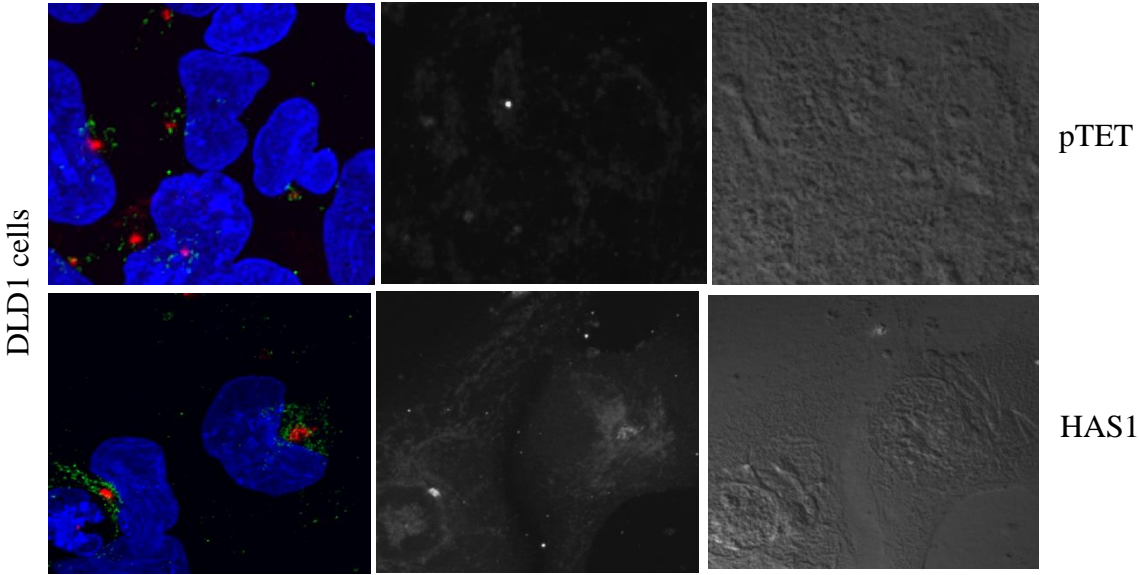

B

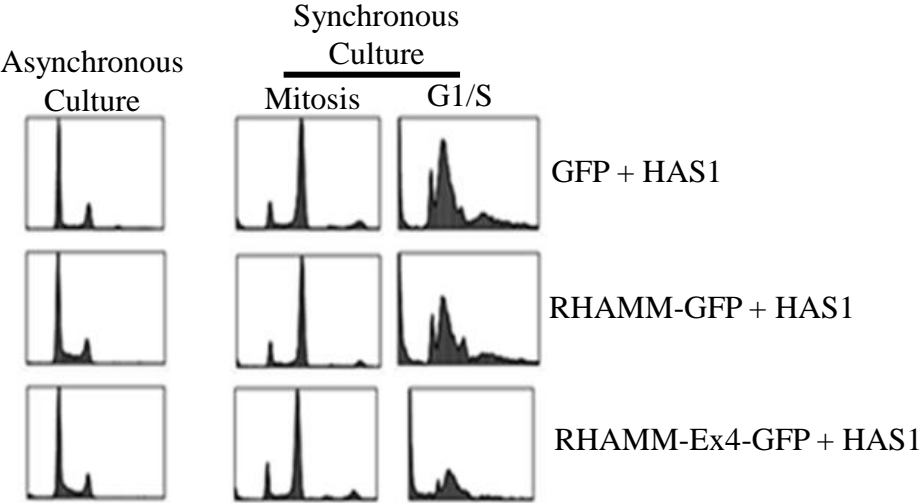

Supplement: Supplementary file 3 — (A) Larger Golgi apparatus were observed in the cells expressing HAS1 (lower panels) as compared to control pTET cells (upper panels). The tetracycline-inducible DLD1 cells with HAS1 and control (pTET) as described in Fig. 5B were stained for Golgi bodies (GM130, green), centrosome (pericentrin, red) and nucleus (blue) in the first panel, and HA (white) in the second panel and DIC image of the structure of the cell in third panel. (B) Respective cell populations indicate the synchronized cells at mitosis and G1/S phase of the cell cycle. Transfected HeLa cells were synchronized with double thymidine blocks. The cells were measured for their DNA contents using flow cytometry to verify synchronization. The cells were harvested, fixed with cold ethanol and stained with propidium iodide to measure the content of DNA in cell-populations. (PDF 158 kb) [file 12964_2017_204_MOESM3_ESM.pdf]
